# A Single Mutation Transforms an Iron Transporter into an Ion Channel

**DOI:** 10.1371/journal.pbio.0020070

**Published:** 2004-03-16

Trace heavy metals are essential for a number of metabolic reactions in living systems, but cells walk a fine line between feast or famine. While iron, zinc, cobalt, and manganese, for example, contribute to the catabolic activity of enzymes involved in essential pathways from gene regulation to cell signaling, even a mild surplus of these metals can kill cells and cause a variety of diseases. Maintaining the proper concentration, or homeostasis, of cellular metals requires strict policing of what passes through cell membranes and organelles.[Fig pbio-0020070-g001]

**Figure pbio-0020070-g001:**
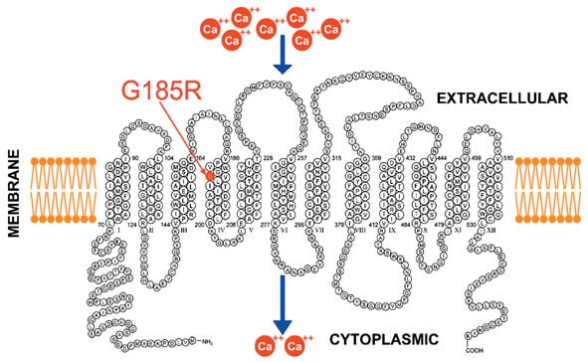
A single mutation of the amino acid glycine (G) to arginine (R) turns a membrane transporter into a calcium channel

One way cells regulate entry is through the hydrophobic lipid (fatty) layer that makes up the cell membrane. While the lipid membrane allows most small fat-soluble or uncharged molecules to simply diffuse through it, nearly all water-soluble molecules, including metal compounds—which typically break down into ions (molecules with positive or negative charge) in solution—rely on either transport or channel proteins to get through.

Two types of proteins manage the transport and uptake of iron ions in mammalian cells: the transferrin receptor helps to concentrate iron in discrete intracellular compartments called endosomes, while a protein called divalent metal transporter-1 (DMT1) releases iron into the cytoplasm, where it supports essential metabolic processes. DMT1 also serves to bring dietary iron directly into the intestinal cells involved in iron absorption. DMT1 preferentially carries iron, zinc, copper, and manganese, but not calcium. This selectivity helps strike the right balance of the concentration of these metals in the cell. Recent structural analyses of transporters, however, have raised the possibility that this selectivity may not be as fixed as once thought. Lending support to the notion that the distinctions between transporters and ion channels are blurring, David Clapham, Nancy Andrews, and colleagues report that a mutation causing a single amino acid substitution in the DMT1 metal ion transporter opens a passageway that converts the transporter into a calcium channel.

DMT1 is essential for maintaining iron homeostasis and the only molecule known to facilitate transmembrane iron uptake in higher eukaryotes, including humans. It is expressed mainly in epithelial cells of the small intestine, where iron metabolism is monitored, and in endosomes, which release transferrin-imported iron. The Clapham and Andrews groups focused on a mutation in the DMT1 transporter called G185R—which substitutes the arginine (R) amino acid for glycine (G) at a particular location in the protein's amino acid chain, position 185—because the identical mutation has occurred spontaneously in three separate laboratory strains of rodents (two mouse and one rat strain). That a single substitution has arisen independently and persisted in multiple rodent generations suggests it may confer some type of selective advantage.

To investigate this idea, the researchers compared the properties of “wild-type” (nonmutant) DMT1 and mutant G185R in laboratory cell lines. They found that cells expressing G185R mutant proteins had much lower levels of iron uptake than cells expressing the nonmutant proteins, but that they also permitted the influx of calcium ions. To see whether the G185R-mediated calcium permeability had a physiological effect on the mice with this mutation, the researchers compared the properties of intestinal epithelial cells taken from the mutant and nonmutant animals. The intestinal cells in the mutant mice showed high levels of the G185R protein and a large current of charged molecules—much as would occur in an ion channel. This current was observed in both the cell lines expressing G185R and the cells extracted from the G185R mutant mice.

The G185R mutation, the researchers conclude, appears to either expose or enhance a calcium “permeation pathway” that exhibits the properties of a calcium channel. This transformation appears to offer a selective advantage, since mice engineered without the DMT1 protein die within a week of birth while mice born with the G185R DMT1 mutation can live for over a year. Though the G185R mice exhibit severe iron deficiency, the modest function retained by G185R in combination with the increased influx of calcium may be enough to extend their lifespan. The increased levels of calcium, the researchers propose, may support iron uptake through some other pathway, an advantage that might explain why such a mutation would persist.

Whatever mechanism accounts for this advantage, the G185R mutation transforms DMT1 transporter into an “unambiguous” calcium ion channel. Investigating the structural and biochemical properties of this molecular changeling will provide valuable insights into the emerging model of a transporter–channel continuum—which suggests a remarkable adaptability to shifting environmental conditions.

